# Interocular Timing Differences in Horizontal Saccades of Ball Game Players

**DOI:** 10.3390/vision9010009

**Published:** 2025-01-31

**Authors:** Masahiro Kokubu, Yoshihiro Komatsu, Takashi Kojima

**Affiliations:** 1 Institute of Health and Sport Sciences, University of Tsukuba, Tsukuba 305-8574, Japan; 2 Graduate School of Comprehensive Human Sciences, University of Tsukuba, Tsukuba 305-8574, Japan

**Keywords:** baseball, binocular eye movements, latency, peak velocity, saccade, soccer

## Abstract

In ball game sports, binocular visual function is important for accurately perceiving the distance of various objects in visual space. However, the temporal coordination of binocular eye movements during saccades has not been investigated extensively in athletes. The purpose of the present study was to compare the characteristics found in the interocular timing differences in horizontal saccades between ball game players. The participants included 32 university baseball players and 54 university soccer players. They were asked to shift their gaze to the onset of the light-emitting diodes located at 10 deg of visual field eccentricity to the left and right and alternated every 2 s. Horizontal movements of the left and right eyes were recorded separately with the electro-oculogram. Temporal variables for each eye were calculated with digital differentiation, and timing differences between the left and right eyes were compared between participant groups. The overall results showed significant interocular differences between left and right eye movements for the temporal variables of binocular saccades. The comparison between the participant groups revealed that baseball players had smaller interocular timing differences between the left and right eyes than soccer players in the onset time, time to peak velocity, duration, and peak velocity. These results suggest that baseball players have a higher degree of temporal coordination in binocular eye movements, particularly during the initial phase of horizontal saccades, compared to soccer players. This enhanced coordination might be attributable to the sport-specific visual demands of baseball, where players require precise stereoscopic vision to track a small high-speed ball within their visual space.

## 1. Introduction

In sports situations, especially in ball games, it is important to quickly shift one’s gaze toward various sources of information, such as the ball, teammates, and opposing players in the visual field in order to plan and execute appropriate movements. Saccadic eye movements, which are rapid movements that position the object image on the central retina, are used to obtain clear visual information from the environment. The properties of saccade in athletes have been examined in numerous studies [1,2,3,4,5,6,7,8,9,10,11]. For instance, athletes such as clay shooters [1], volleyball players [9,11], and some open-skill sports athletes [2] have shorter saccade latencies compared to nonathletes. Similarly, soccer players have higher average saccade velocity [12,13]. Thus, saccades are considered to play an important role in athletes.

For saccades, binocular coordination is considered essential for achieving clear binocular vision [14,15]. For example, binocular fusion is difficult in conditions such as amblyopia or strabismus, where the eyes do not properly align with each other when looking at an object [16,17]. Binocular vision provides information that is necessary for stereopsis, including depth cues to perceive the distance of an object. In sports contexts, binocular information about time to collision is more effective than monocular information [18]. However, the issue we need to consider here is that research on the binocular function of athletes has been insufficient despite its assumed importance in sports. Although it has been recently suggested that binocular vision function plays an important role in the motor skills of ball game athletes, including soccer, basketball, and handball players [19,20,21], binocular coordination during saccades in athletes remains unexamined. Therefore, the present study focused on the binocular coordination of saccades in athletes.

Binocular eye movements can be classified into two types: conjugate eye movements, in which both eyes move in the same direction, and non-conjugate eye movements, in which both eyes move in the opposite direction [22]. In previous studies focusing on sports situations and athletes, saccades have been measured in only one eye (i.e., left or right eye) or in both eyes together, meaning that the conjugacy of binocular saccades has not been examined. This is likely to be due to the assumption that the left and right eyes make the same movements during saccades. However, several studies on human participants have demonstrated slight but significant differences in latency and velocity between the two eyes [23,24]. Additionally, it has been suggested that horizontal saccades include not only a conjugate component but also a vergence component in regard to eye movements [22,24,25,26]. Therefore, it is important to examine the coordination of binocular eye movements in athletes. The present study measured and analyzed saccades of the left and right eyes separately to investigate the characteristics of binocular coordination in athletes.

The visual skills that are important for performance are specific to the type of ball game. For instance, stereopsis may be more critical in certain sports than in others. A review of the literature suggests that interceptive sports such as baseball and cricket rely heavily on dynamic stereopsis for actions like catching or intercepting, whereas strategic sports such as soccer, football, hockey, basketball, and volleyball rely more on other visual skills, including peripheral and spatial vision [27]. Baseball players are required to track a small high-speed ball with their eyes, which likely requires superior stereoscopic visual functions. On the other hand, soccer players need to focus on multiple moving objects such as teammates, opponents, and the ball in a broader and more dynamic visual environment. Considering these differences, it is postulated that baseball players are likely to exhibit superior coordination of the two eyes compared to soccer players.

To investigate this hypothesis, we compared the interocular temporal differences observed in the saccades of baseball and soccer players. The purpose of the present study was to compare the characteristics of the interocular timing differences in horizontal saccades between ball game players. We hypothesized that baseball players would exhibit smaller temporal differences between the two eyes than soccer players.

## 2. Materials and Methods

### 2.1. Participants

The participants were 32 university baseball players (age = 19.8 ± 1.3 years; years of baseball experience = 13.2 ± 1.9 years; mean ± SD) and 54 university soccer players (age: 20.3 ± 1.4 years; years of soccer experience = 13.2 ± 2.4 years). The participants’ teams belonged to the top league in their district and had previously won a national championship for college students. All participants had normal or corrected-to-normal visual acuity, and none of them had any known neuromuscular disorders.

Prior to the measurement, they were informed of the experimental protocols and provided their written informed consent to participate in the study. The protocol for the present study was approved by the Ethics Committee of the Institute of Health and Sport Science, University of Tsukuba. All procedures were conducted in accordance with the principles of the Declaration of Helsinki.

### 2.2. Apparatus

The participants were seated in front of a horizontal table. Their heads were stabilized with an adjustable chin rest and head support to prevent the vestibular system from affecting their eye movements. The participants’ eye level was positioned 38 cm above the table.

**Figure 1:**
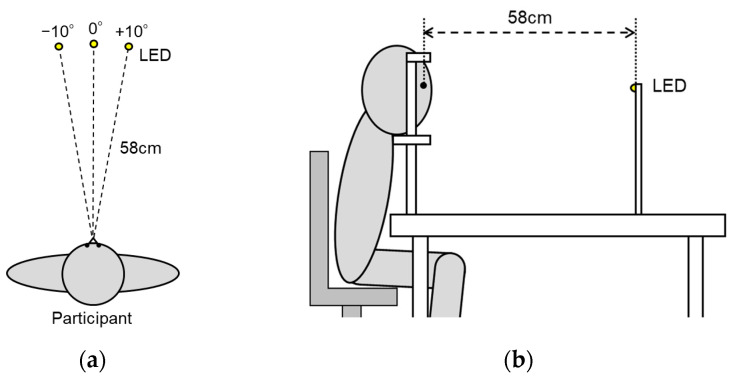
Positions of the visual stimulus LEDs: (**a**) Top view; (**b**) Side view.

The experimental setup is illustrated in Figure 1. Visual stimuli were presented by light-emitting diodes (LEDs). The LEDs were 5 mm in diameter and emitted yellow light (590 nm). They were mounted on top of an aluminum frame aligned with eye level and positioned at a viewing distance of 58 cm from the center of the participants’ eyes, with an eccentricity of 10 deg to the left and right. The illumination pattern of the LEDs was controlled and presented using LabChart8 and PowerLab 8/35 (ADInstruments Japan, Tokyo, Japan).

**Figure 2:**
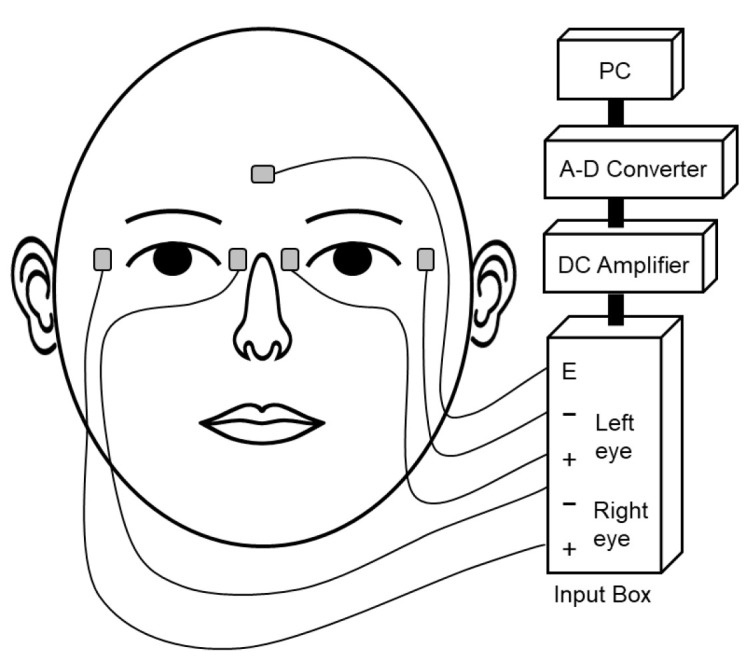
EOG electrode placements and recording systems.

Electro-oculogram (EOG) was used to measure eye movements. Ag-AgCl disposable small bioelectrodes (N-03JS3, Nihon Kohden, Tokyo, Japan) were attached near the lateral and medial canthus of each eye and on the forehead as a reference electrode (Figure 2). The EOG signal of each eye was amplified with two DC amplifiers (AN-601G, Nihon Kohden, Tokyo, Japan), and the horizontal component of each eye was recorded separately at 1000 Hz using an analog-to-digital converter (PowerLab 8/35). It is known that the relation between the EOG potential and the angular position of the eye is linear within the range of ±15–20 deg [28,29,30,31]. Thus, in the present study, the eccentricity of 10 deg was chosen for positioning the LEDs, as it ensured the linearity of the EOG.

### 2.3. Procedure

The participants wore bioelectrodes, and their heads were fixed in place using a chin rest and head support. Before the actual trials began, EOG calibration was performed for each participant. The participants were instructed to direct their eyes towards three LEDs located at −10, 0, and 10 deg every 2 s. The change in EOG potential during eye movement was recorded as reference values. The angular position of the eye was calculated using linear regression based on the EOG potential.

During the actual trials, the participants performed gaze shifts to the left and right toward the alternating LEDs every 2 s. The reason for setting the time interval between trials to 2 s was to reduce the likelihood of blinking just before or during the gaze shift. They were instructed to shift their gaze as quickly as possible after each LED was illuminated. Although the timing of the LED illumination was predictable in this task, participants were asked not to make predictive saccades prior to the illumination. Participants were also instructed not to blink during the gaze movements. Each participant performed 20 trials in total, with 10 trials in each direction.

### 2.4. Data Analysis

**Figure 3:**
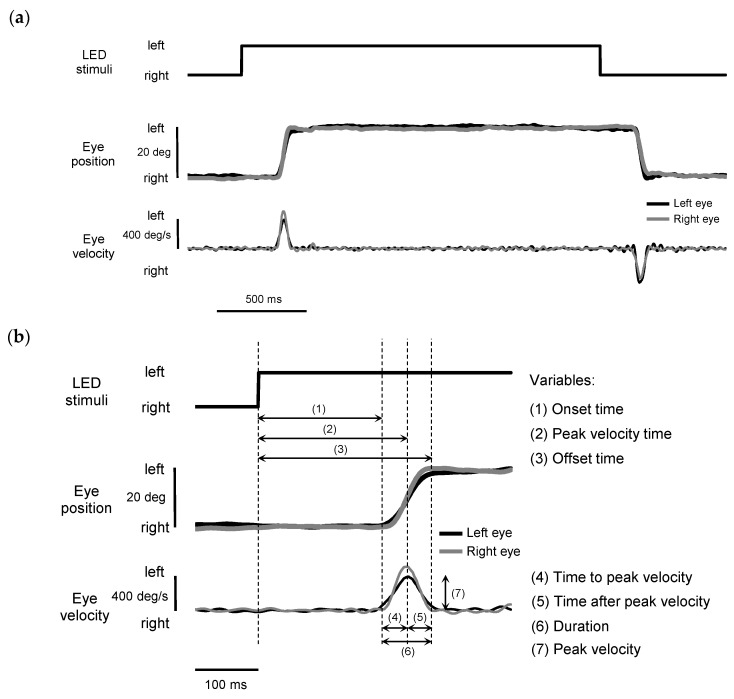
Typical examples of the horizontal position (bold lines) and velocity (thin lines) of binocular saccades in response to LED stimuli. Black and gray lines indicate the left and right eye, respectively. (**a**) The time-series data recorded during saccades to the left and right LEDs. (**b**) Definitions of saccade variables analyzed in the present study. The dashed vertical lines represent key temporal events in the response. Please note that, for the sake of simplicity, the labels displayed with each arrow are derived exclusively from the left eye’s data.

Typical examples of the horizontal position and velocity of binocular saccades in response to LED stimuli are shown in Figure 3. The time-series data for binocular saccades to the left and right LEDs are presented in Figure 3a. During off-line data analysis, saccade variables were calculated and identified with a custom MATLAB script (MATLAB 2023a, MathWorks Inc, Natick, MA, USA). The horizontal position of each eye was filtered using a low-pass filter with a cutoff frequency of 30 Hz and a high-pass filter with a cutoff frequency at 0.05 Hz [32]. A 50 Hz notch filter was applied to suppress line noise. The filtered data were used to obtain eye velocity using a five-point differentiation algorithm. The resulting velocity profile of each eye was analyzed to determine the initiation and termination points of saccades.

The saccade variables analyzed in the present study are depicted in Figure 3b. Peak velocity (deg/s) was defined as the maximum eye velocity attained during horizontal saccade. The initiation and termination of the horizontal saccade were detected by a threshold of 10% of the peak velocity; practically, this corresponded to 40 deg/s (as the peak velocity of 20 deg saccades is typically above 400 deg/s). These criteria are consistent with those used by other authors [33,34]. Onset time, peak velocity time, and offset time were defined as the time periods from the illumination of the stimulus LED to the moments of the initiation, peak velocity, and termination of saccade, respectively. Time to peak velocity was defined as the interval between onset time and peak velocity time. The time after peak velocity was defined as the interval between the peak velocity time and the offset time. Duration was defined as the interval between the onset time and offset time.

We excluded trials from the analysis where the onset time was less than 100 ms due to anticipation or more than 1000 ms due to lack of attention. In addition, trials in which the participant blinked during the period between the stimulus LED onset and the initiation of the saccade were eliminated from further analyses. Trials in which saccades could not be properly detected were also excluded. In total, 97 trials (5.6% of total trials) were excluded based on these criteria.

### 2.5. Statistics

The mean values for the saccade variables mentioned above were calculated for each direction and each eye. We included seven dependent variables in the analysis: onset time, peak velocity time, offset time, time to peak velocity, time after peak velocity, duration, and peak velocity.

We conducted a multivariate analysis of variance (MANOVA) referring to the statistical framework used by Mouga et al. [35]. A MANOVA with a three-way interaction was used to evaluate differences in the saccade variables by group (baseball, soccer), direction (left, right), and eye (left, right). A multivariate approach was adopted because of its independence from sphericity assumptions. The goal of the three-way MANOVA was to understand if there was an interaction effect for the group, direction, and eye in the saccade variables. Follow-up univariate three-way ANOVAs were run for each dependent variable. For dependent variables with statistically significant interaction effects, simple two-way interactions and main effects of the group were conducted. Partial eta-squared (*η*_p_^2^) values were reported as a measure of effect size. All statistical analyses were completed with SPSS version 28 (Chicago, IL, USA). The general significance threshold was set to an error probability of *p* < 0.05.

## 3. Results

### 3.1. MANOVA and ANOVA for All Dependent Variables

The saccade variables for baseball and soccer players are presented in Table 1. A three-way MANOVA was conducted with seven eye movement-related dependent variables (onset time, peak velocity time, offset time, time to peak velocity, time after peak velocity, duration, and peak velocity) and three independent variables (direction (leftward and rightward), eye (left and right), and group (baseball and soccer)). There was a statistically significant three-way interaction between direction, eye, and group in all dependent variables together, including Pillai’s Trace = 0.129; *F*(4, 81) = 3.006, *p* = 0.023, *η*_p_^2^ = 0.129, and observed power = 0.777 (Table S1).

Follow-up univariate three-way ANOVAs were performed for each dependent variable. These showed a statistically significant three-way interaction effect of direction × eye × group for onset time [*F*(1, 84) = 8.497, *p* = 0.005, *η*_p_^2^ = 0.092, observed power = 0.822], time to peak velocity [*F*(1, 84) = 7.514, *p* = 0.007, *η*_p_^2^ = 0.082, observed power = 0.773], duration [*F*(1, 84) = 8.060, *p* = 0.006, *η*_p_^2^ = 0.088, observed power = 0.801], and peak velocity [*F*(1, 84) = 8.958, *p* = 0.004, *η*_p_^2^ = 0.096, observed power = 0.841], but not for peak velocity time [*F*(1, 84) = 0.364, *p* = 0.548, *η*_p_^2^ = 0.004, observed power = 0.092], offset time [*F*(1, 84) = 0.957, *p* = 0.331, *η*_p_^2^ = 0.011, observed power = 0.162] and time after peak velocity [*F*(1, 84) = 0.593, *p* = 0.443, *η*_p_^2^ = 0.007, observed power = 0.119]. A significant interaction effect of direction × eye was observed for peak velocity time [*F*(1, 84) = 7.376, *p* = 0.008, *η*_p_^2^ = 0.081, observed power = 0.766], offset time [*F*(1, 84) = 13.104, *p* < 0.001, *η*_p_^2^ = 0.135, observed power = 0.947], and time after peak velocity [*F*(1, 84) = 6.283, *p* = 0.014, *η*_p_^2^ = 0.070, observed power = 0.698], indicating that interocular differences depended on the direction of the saccade but did not differ between groups for these variables.

**Figure 4:**
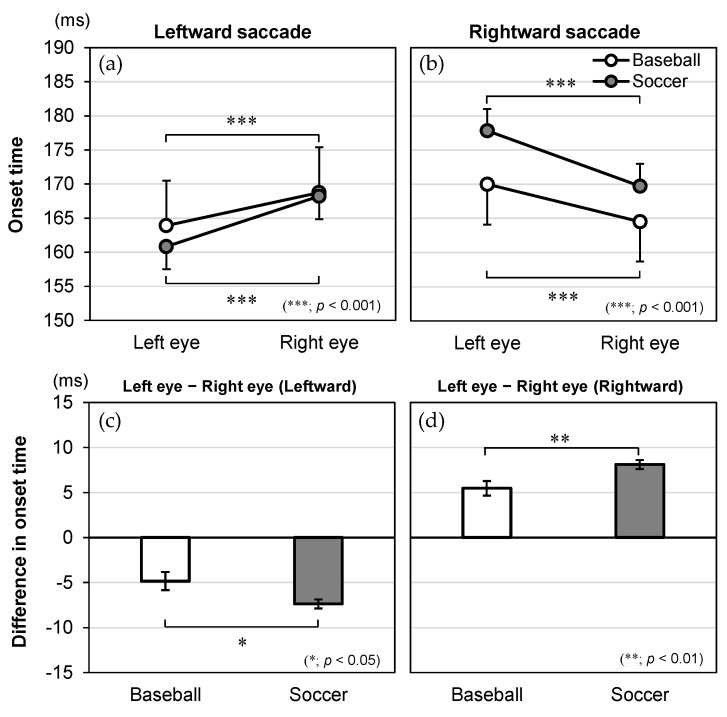
Interaction effects between group, eye, and direction for onset time (ms). The onset time is shown for the eye (left and right), plotted by group (baseball and soccer) in the (**a**) leftward and (**b**) rightward saccades. The difference in onset time (left eye-right eye) is shown for the group (baseball and soccer) in the (**c**) leftward and (**d**) rightward saccades. Data are expressed as mean and standard error.

**Figure 5:**
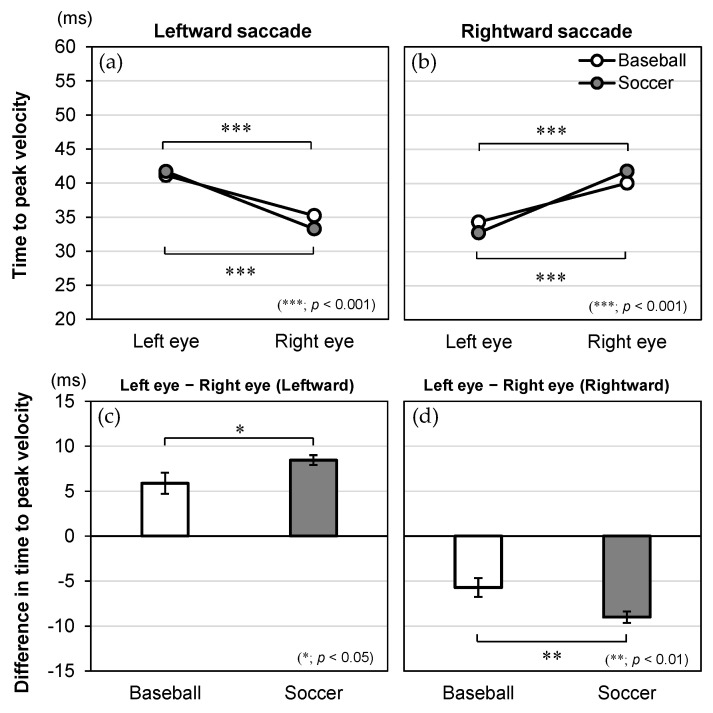
Interaction effects between the group, eye, and direction in regard to the time to peak velocity (ms). The time to peak velocity is shown for the eye (left and right) and plotted by group (baseball and soccer) in the (**a**) leftward and (**b**) rightward saccades. The difference in the time to peak velocity (left eye-right eye) is shown for the group (baseball and soccer) in the (**c**) leftward and (**d**) rightward saccades. The data are expressed as mean and standard error.

**Figure 6:**
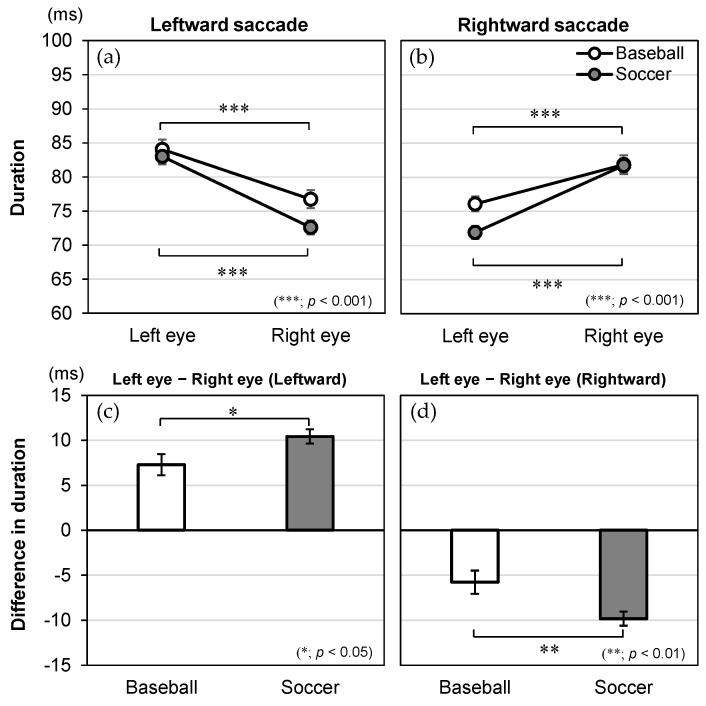
Interaction effects between the group, eye, and direction in regard to duration (ms). Duration is shown for the eye (left and right) and plotted by group (baseball and soccer) in the (**a**) leftward and (**b**) rightward saccades. The difference in duration (left eye-right eye) is shown for the group (baseball and soccer) in the (**c**) leftward and (**d**) rightward saccades. The data are expressed as mean and standard error.

**Figure 7:**
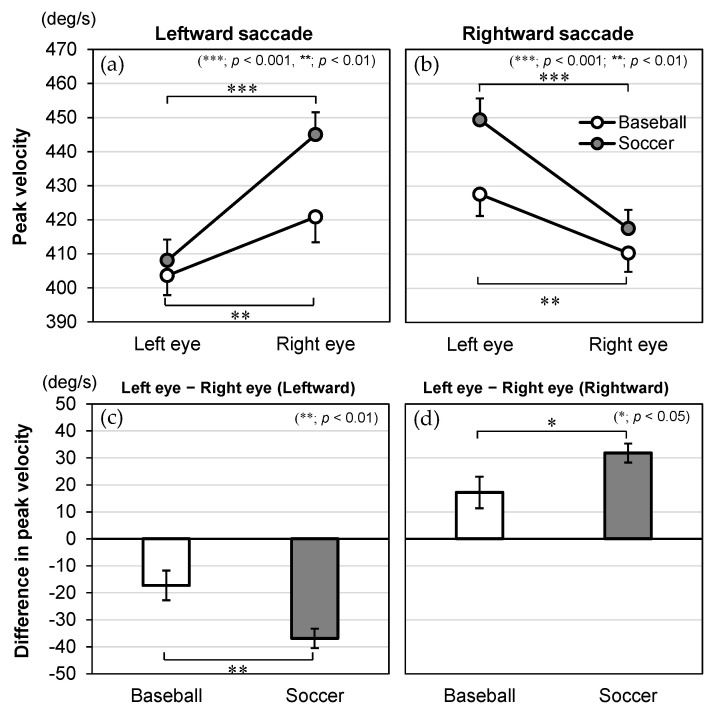
Interaction effects between the group, eye, and direction in regard to the peak velocity (deg/s). Peak velocity is shown for the eye (left and right) and plotted by group (baseball and soccer) in the (**a**) leftward and (**b**) rightward saccades. The difference in peak velocity (left eye-right eye) is shown for the group (baseball and soccer) in the (**c**) leftward and (**d**) rightward saccades. The data are expressed as mean and standard error.

The significant interaction effects of direction × eye × group for onset time, time to peak velocity, duration, and peak velocity are illustrated in Figure 4, Figure 5, Figure 6 and Figure 7, respectively. For these dependent variables with statistically significant three-way interaction effects, simple two-way ANOVAs were performed to examine the main effects of the eye and group and their interaction effects for saccades in each direction.

### 3.2. Onset Time

As for onset time (Figure 4), the two-way ANOVA revealed a significant main effect for eye [*F*(1, 84) = 146.938, *p* < 0.001, *η*_p_^2^ = 0.636 for leftward; *F*(1, 84) = 227.723, *p* < 0.001, *η*_p_^2^ = 0.731 for rightward] but not for group [*F*(1, 84) = 0.075, *p* = 0.785, *η*_p_^2^ = 0.001 for leftward; *F*(1, 84) = 1.129, *p* = 0.291, *η*_p_^2^ = 0.013 for rightward]. There was also a significant interaction effect of eye × group [*F*(1, 84) = 6.259, *p* = 0.014, *η*_p_^2^ = 0.069 for leftward; *F*(1, 84) = 8.577, *p* = 0.004, *η*_p_^2^ = 0.093 for rightward].

Post hoc tests to evaluate the interaction effect showed that the simple main effect of eye was significant in both groups. For the leftward saccade, the left eye showed a shorter onset time than the right eye in baseball players (Mean ± SE, left eye: 163.9 ± 6.6 ms, right eye: 168.8 ± 6.6 ms, *p* < 0.001) and soccer players (left eye: 160.8 ± 3.3 ms, right eye: 168.2 ± 3.4 ms, *p* < 0.001). For the rightward saccade, the right eye showed a shorter onset time than theleft eye in baseball players (left eye: 170.0 ± 5.9 ms, right eye: 164.5 ± 5.8 ms, *p* < 0.001) and soccer players (left eye: 177.8 ± 3.2 ms, right eye: 169.7 ± 3.2 ms, *p* < 0.001). Importantly, soccer players had a larger absolute difference between the left and right eyes than baseball players for the leftward (baseball: −4.8 ± 1.0 ms, soccer: −7.4 ± 0.5 ms, *p* = 0.014) and rightward (baseball: 5.5 ± 0.8 ms, soccer: 8.1 ± 0.5 ms, *p* = 0.004) saccades.

### 3.3. Time to Peak Velocity

As for time to peak velocity (Figure 5), the two-way ANOVA revealed a significant main effect for eye [*F*(1, 84) = 156.447, *p* < 0.001, *η*_p_^2^ = 0.651 for leftward; *F*(1, 84) = 160.582, *p* < 0.001, *η*_p_^2^ = 0.657 for rightward] but not for group [*F*(1, 84) = 1.019, *p* = 0.316, *η*_p_^2^ = 0.012 for leftward; *F*(1, 84) = 0.030, *p* = 0.863, *η*_p_^2^ = 0.000 for rightward]. There was also a significant interaction effect of eye × group [*F*(1, 84) = 5.080, *p* = 0.027, *η*_p_^2^ = 0.057 for leftward; *F*(1, 84) = 8.060, *p* = 0.006, *η*_p_^2^ = 0.088 for rightward].

Post hoc tests to evaluate the interaction effect showed that the simple main effect of eye was significant in both groups. For the leftward saccade, the left eye showed a longer time to peak velocity than the right eye in baseball players (left eye: 41.1 ± 0.9 ms, right eye: 35.2 ± 0.7 ms, *p* < 0.001) and soccer players (left eye: 41.7 ± 0.6 ms, right eye: 33.3 ± 0.4 ms, *p* < 0.001). For the rightward saccade, the right eye showed a longer time to peak velocity than the left eye in baseball players (left eye: 34.3 ± 0.5 ms, right eye: 40.0 ± 0.8 ms, *p* < 0.001) and soccer players (left eye: 32.8 ± 0.4 ms, right eye: 41.8 ± 0.6 ms, *p* < 0.001). Importantly, soccer players had a larger absolute difference between left and right eyes than baseball players for the leftward (baseball: 5.9 ± 6.7 ms, soccer: 8.5 ± 4.0 ms, *p* = 0.027) and rightward (baseball: −5.7 ± 5.9 ms, soccer: −9.0 ± 4.7 ms, *p* = 0.006) saccades.

### 3.4. Duration

As for duration (Figure 6), the two-way ANOVA revealed a significant main effect for eye [*F*(1, 84) = 165.359, *p* < 0.001, *η*_p_^2^ = 0.663 for leftward; *F*(1, 84) = 121.285, *p* < 0.001, *η*_p_^2^ = 0.591 for rightward] but not for group [*F*(1, 84) = 2.535, *p* = 0.115, *η*_p_^2^ = 0.029 for leftward; *F*(1, 84) = 2.241, *p* = 0.138, *η*_p_^2^ = 0.026 for rightward]. There was also a significant interaction effect of eye × group [*F*(1, 84) = 5.166, *p* = 0.026, *η*_p_^2^ = 0.058 for leftward; *F*(1, 84) = 8.207, *p* = 0.005, *η*_p_^2^ = 0.089 for rightward].

The post hoc tests used to evaluate the interaction effect showed that the simple main effect of eye was significant in both groups. For the leftward saccade, the left eye showed a longer duration than the right eye in baseball players (left eye: 84.0 ± 1.5 ms, right eye: 76.7 ± 1.3 ms, *p* < 0.001) and soccer players (left eye: 83.0 ± 1.1 ms, right eye: 72.6 ± 1.0 ms, *p* < 0.001). For the rightward saccade, the right eye showed a longer duration than the left eye in baseball players (left eye: 76.1 ± 1.1 ms, right eye: 81.8 ± 1.4 ms, *p* < 0.001) and soccer players (left eye: 71.9 ± 1.0 ms, right eye: 81.7 ± 1.0 ms, *p* < 0.001). Importantly, soccer players had a larger absolute difference between the left and right eyes than baseball players for the leftward (baseball: 7.3 ± 1.2 ms, soccer: 10.4 ± 0.8 ms, *p* = 0.026) and rightward (baseball: −5.8 ± 1.3 ms, soccer: −9.8 ± 0.8 ms, *p* = 0.005) saccades.

### 3.5. Peak Velocity

As for peak velocity (Figure 7), the two-way ANOVA revealed a significant main effect for eye [*F*(1, 84) = 73.343, *p* < 0.001, *η*_p_^2^ = 0.466 for leftward; *F*(1, 84) = 57.756, *p* < 0.001, *η*_p_^2^ = 0.407 for rightward] but bot for group [*F*(1, 84) = 2.446, *p* = 0.122, *η*_p_^2^ = 0.028 for leftward; *F*(1, 84) = 2.883, *p* = 0.093, *η*_p_^2^ = 0.033 for rightward]. There was also a significant interaction effect of eye × group [*F*(1, 84) = 9.660, *p* = 0.003, *η*_p_^2^ = 0.103 for leftward; *F*(1, 84) = 5.108, *p* = 0.026, *η*_p_^2^ = 0.057 for rightward].

The post hoc tests used to evaluate the interaction effect showed that the simple main effect of eye was significant in both groups. For the leftward saccade, the peak velocity of the right eye was higher compared to the left eye in baseball players (left eye: 403.6 ± 5.7 deg/s, right eye: 420.8 ± 7.4 deg/s, *p* = 0.004) and soccer players (left eye: 408.1 ± 6.1 deg/s, right eye: 445.0 ± 6.6 deg/s, *p* < 0.001). For the rightward saccade, the peak velocity of the left eye was higher compared to the right eye in baseball players (left eye: 427.5 ± 6.1 deg/s, right eye: 410.3 ± 6.8 deg/s, *p* = 0.006) and soccer players (left eye: 449.4 ± 6.3 deg/s, right eye: 417.6 ± 5.5 deg/s, *p* < 0.001). Importantly, soccer players had a larger absolute difference between the left and right eyes than baseball players for leftward (baseball: −17.2 ± 5.5 deg/s, soccer: −36.9 ± 3.6 deg/s, *p* = 0.003) and rightward (baseball: 17.2 ± 5.9 deg/s, soccer: 31.8 ± 3.5 deg/s, *p* = 0.026) saccades.

## 4. Discussion

### 4.1. Interocular Timing Differences and Sport-Specific Demands

The purpose of the present study was to examine interocular timing differences in the horizontal saccades between baseball and soccer players. The overall results indicated significant timing differences between the left and right eye movements in the temporal variables of binocular saccades. The comparison of the two participant groups revealed distinct differences in binocular coordination. Baseball players exhibited smaller differences in temporal variables between their left and right eyes compared to soccer players. These variables included onset time, time to peak velocity, duration, and peak velocity, all of which are important for assessing the coordination of binocular saccades. The implications of the results are discussed in terms of sport-specific visual demands on binocular coordination.

Previous findings have provided quantitative evidence of domain-specific visual expertise in athletes of interceptive sports and strategic sports [27,36]. Baseball is classified as one of the interceptive sports. In this sport, visual functions, such as depth perception and distance stereoacuity, are considered important for skills such as hitting and catching [37,38]. Some comparative studies have shown that youth baseball and softball players have better static stereoacuity [39] and a greater ability to track moving objects with their eyes [40] than non-players. Baseball players’ superior temporal coordination between the two eyes might stem from the visual demands of tracking a small high-speed ball in space, which necessitates precise binocular synchronization. These requirements likely promote the development of binocular coordination in baseball players through long-term practice.

In addition, the role of vergence in baseball players and the interaction between saccades and vergence have been considered. An earlier study revealed that baseball players had shorter latency in convergence eye movements compared to nonathletes, suggesting that visual experience and baseball training could influence the gaze shift dynamics during convergence [10]. It has also been shown that saccade latency decreases and binocular coordination improves after vergence rehabilitation, highlighting the interaction between saccades and vergence [41]. Based on these findings, it is possible that baseball players improve binocular coordination by enhancing vergence through daily training.

Conversely, other studies suggest that eye movements do not contribute significantly to baseball skills. For example, there were no significant differences between baseball players and nonathletes in terms of saccade onset or offset latency [42]. In an actual baseball hitting task, the tracking motion of the head became faster as the launched ball came close to the hitters, while there was no change in the angular velocity of eye movement, suggesting that rapid eye movements would not be used during fastball hitting in baseball [43]. In these previous studies, however, the contribution of binocular coordination was not considered. The results of our study suggest that binocular coordination may be more critical for baseball performance than the latency and velocity of eye movements.

Soccer, on the other hand, is classified as a strategic sport [27,36]. Soccer players operate in a broader and more dynamic visual environment, requiring them to monitor multiple moving objects, such as teammates, opponents, and the ball [44,45,46,47]. A soccer player’s visual focus is often distributed across a larger field area with “zoom out” operations rather than local attending on a single small object [48]. These visual demands may explain the larger interocular timing differences observed in soccer players, as their oculomotor system prioritizes flexibility and rapid shifts in attention over precise binocular coordination and stereoscopic visual function relative to baseball players.

In fact, earlier studies have reported that professional and amateur soccer players do not show superior stereoscopic performance compared to inexperienced participants [49] and that 36.4% of elite soccer players experience binocular vision dysfunction [50]. Furthermore, it has been shown that soccer players with worse stereopsis in distance vision tend to exhibit lower dynamic visual acuity [51]. Previous studies have suggested that dynamic visual acuity is related to distinct eye movement patterns [40,52]. Taking these findings into consideration, it is possible that soccer players do not have superior binocular coordination of saccades compared to baseball players.

It should be noted, however, that soccer players are superior in terms of visual reaction and saccade parameters. Some studies have shown that soccer players demonstrate significantly shorter choice reaction times for monocular stimuli than inexperienced participants [49]. Additionally, skilled soccer players show shorter latency and smaller variability in saccade latency than their less skilled counterparts [53] and higher saccade velocity than nonathletes [12,13].

### 4.2. Temporal Asymmetry Between the Abducting and Adducting Eyes

The overall results demonstrated temporal asymmetries in saccade dynamics between the abducting eye (i.e., moving temporally) and the adducting eye (i.e., moving nasally) for both participant groups. For leftward saccades, the left eye showed a shorter onset time than the right eye, while, for rightward saccades, the right eye showed a shorter onset time than the left eye. Thus, across both groups, the abducting eye exhibited a shorter onset time than the adducting eye. The asymmetry observed in the present study is consistent with the results of earlier studies, which demonstrated a slight yet significant difference in saccade latency between the two eyes [23,24,54]. Our results suggest that horizontal saccades involve not only a conjugate component but also a disjunctive (i.e., vergence) component of the eyes, reflecting the subtle adjustments necessary to maintain binocular alignment, as seen in the earlier studies [22,24,25,26]. One possible explanation for the interocular timing difference during horizontal saccade is the presence of the additional synaptic delay caused by the abducens internuclear neuron in the pathway to the medial rectus muscle of the eye [23].

As for peak velocity, the adducting eye exhibited a higher value than the abducting eye for both participant groups. This result is partly consistent with previous studies showing that the peak velocity of the adducting eye is faster than that of the abducting eye [54]. However, other studies indicate that peak velocity is faster in the abducting eye than in the adducting eye [24,55], highlighting inconsistencies across studies. In addition, the present results showed shorter duration in the adducting eye than in the abducting eye. These findings differ from an earlier study that reported a consistent delay of about 1 ms in the adducting eye compared to the abducting eye across all horizontal components of saccades [23]. Although the reason for the discrepancies between studies cannot be specified, the differences may partly reflect variations in participant groups. While previous studies measured binocular eye movements in normal human participants, the present study focused on athletes. As athletes regularly practice aligning both eyes quickly on a target, it is possible that the eye with a later onset may catch up with the eye with an earlier onset, resulting in a higher peak velocity and shorter duration.

The significant interaction effect of direction × eye was also observed for peak velocity time, offset time, and time after peak velocity, indicating that the temporal difference between the left and right eyes was dependent on the direction of saccade. However, the interaction effect of direction × eye × group was not significant for these variables. The results indicate no difference between baseball and soccer players in the magnitude of temporal asymmetry between the two eyes in the deceleration phase of the saccade. It is speculated that the difference between sports regarding asymmetry in binocular eye movement appears in the acceleration phase of eye movement rather than in the deceleration phase.

### 4.3. Implications for Binocular Vision in Sports and Practical Applications

The findings of the present study have important implications for understanding the role of binocular vision in sports performance. Binocular vision provides critical information for stereopsis, depth perception, and time-to-collision judgments, all of which are essential for successful performance in catching [56,57,58], hitting [18], and both skills [38]. Furthermore, binocular coordination contributes to various perceptual-cognitive skills in both daily life and sports situations. For example, binocular saccade coordination plays an important role in reading skills [59,60]. It is also known that binocular coordination improves with growth, as adults demonstrate greater coordination than children [14,61,62]. These developmental improvements highlight the importance of understanding how binocular vision evolves over time and its implications for enhancing sports performance.

The present study extends previous findings by suggesting that binocular coordination is also a key component of superior athletic performance, particularly in sports that demand precise tracking of small, fast-moving objects, such as baseball. The differences observed between baseball and soccer players highlight the need to consider sport-specific visual demands when designing training programs for athletes. For baseball players, drills that emphasize fine binocular coordination, such as tracking small objects at high speeds or engaging in tasks that require precise depth perception, may be particularly beneficial. Moreover, research suggests that spatiotemporal coupling in the early visual pathway builds on the information dynamics of the oculomotor cycle rather than initiating coarse-to-fine processing [63]. This spatiotemporal coupling highlights the importance of training that integrates both spatial and temporal visual skills. For soccer players, exercises that enhance broader visual awareness and rapid attention shifts across the field may be more relevant. Incorporating sport-specific vision training into athletic development programs could help optimize performance and reduce the risk of errors in critical situations.

### 4.4. Limitations

While the present study provides original insights into the binocular coordination of athletes, some methodological limitations should be noted. First, the sample was limited to university-level players, which may not fully represent the range of visual and oculomotor abilities across different age groups and skill levels. Future research should include not only professional athletes, whose advanced experience and training might reveal unique patterns, but also younger athletes, to explore how binocular coordination develops from early stages to elite performance. This should also consider the emerging development of visuomotor control, specifically in terms relevant to sports, such as reaching and grasping behaviors in children [64], as these behaviors provide key insights into the foundational processes underlying sports-specific motor skills. Expanding the participant pool to include a broader range of athletes would provide a more comprehensive understanding of interocular timing differences and their role in athletic performance.

Second, the experimental task involved predictable leftward and rightward saccades to fixed targets, which may not fully capture the complexity of real-world sports scenarios. In actual game situations, athletes are required to perform saccades in response to unpredictable and dynamic stimuli, which may place differing demands on their oculomotor systems. It would be beneficial for future studies to incorporate more ecologically valid tasks, such as tracking a moving ball or responding to sudden changes in visual stimuli, to gain deeper insights into how binocular coordination functions in real-world contexts.

## 5. Conclusions

The present study aimed to compare the characteristics of interocular timing differences in horizontal saccades between ball game athletes with distinct visual demands. Baseball and soccer players were asked to shift their gaze with horizontal saccades. The horizontal movements of the left and right eyes were recorded separately with the electro-oculogram. The results showed significant differences in binocular coordination, with baseball players exhibiting smaller interocular timing differences compared to soccer players, particularly in parameters such as onset time, time to peak velocity, duration, and peak velocity. These findings suggest that baseball players have a higher degree of temporal coordination in binocular eye movements, particularly during the initial phase of horizontal saccades, compared to soccer players. The specific visual demands of their respective sports may influence the development of oculomotor skills. This is the first study to demonstrate that interocular timing differences in horizontal saccades vary depending on the sport.

## Figures and Tables

**Table 1:** The comparison of the mean ± SE of saccade variables between baseball and soccer players.

Variables	Direction	Baseball Group			Soccer Group			
		Left Eye	Right Eye	Difference (Left − Right)	Left Eye	Right Eye	Difference (Left − Right)	
		Mean ± SE	Mean ± SE	Mean ± SE	Mean ± SE	Mean ± SE	Mean ± SE	
Onset time (ms)	Leftward	163.9 ± 6.6	168.8 ± 6.6	−4.8 ± 1.0	160.8 ± 3.3	168.2 ± 3.4	−7.4 ± 0.5	*
	Rightward	170.0 ± 5.9	164.5 ± 5.8	5.5 ± 0.8	177.8 ± 3.2	169.7 ± 3.2	8.1 ± 0.5	**
Peak velocity time (ms)	Leftward	205.0 ± 6.7	204.0 ± 6.5	1.0 ± 0.6	202.6 ± 3.4	201.5 ± 3.4	1.1 ± 0.4	
	Rightward	204.3 ± 5.8	204.6 ± 5.9	−0.2 ± 0.5	210.6 ± 3.3	211.5 ± 3.2	−0.9 ± 0.4	
Offset time (ms)	Leftward	247.9 ± 6.7	245.5 ± 6.8	2.4 ± 0.9	243.8 ± 3.6	240.8 ± 3.7	3.1 ± 0.7	
	Rightward	246.1 ± 6.0	246.4 ± 6.0	−0.3 ± 1.0	249.8 ± 3.5	251.5 ± 3.5	−1.7 ± 0.7	
Time to peak velocity (ms)	Leftward	41.1 ± 0.9	35.2 ± 0.7	5.9 ± 1.2	41.7 ± 0.6	33.3 ± 0.4	8.5 ± 0.5	*
	Rightward	34.3 ± 0.5	40.0 ± 0.8	−5.7 ± 1.0	32.8 ± 0.4	41.8 ± 0.6	−9.0 ± 0.6	**
Time after peak velocity (ms)	Leftward	42.9 ± 0.9	41.5 ± 1.0	1.4 ± 0.7	41.3 ± 0.8	39.3 ± 0.8	2.0 ± 0.7	
	Rightward	41.8 ± 0.9	41.8 ± 0.8	−0.1 ± 0.8	39.1 ± 0.7	40.0 ± 0.7	−0.8 ± 0.5	
Duration (ms)	Leftward	84.0 ± 1.5	76.7 ± 1.3	7.3 ± 1.2	83.0 ± 1.1	72.6 ± 1.0	10.4 ± 0.8	*
	Rightward	76.1 ± 1.1	81.8 ± 1.4	−5.8 ± 1.3	71.9 ± 1.0	81.7 ± 1.0	−9.8 ± 0.8	**
Peak velocity (deg/s)	Leftward	403.6 ± 5.7	420.8 ± 7.4	−17.2 ± 5.5	408.1 ± 6.1	445.0 ± 6.6	−36.9 ± 3.6	**
	Rightward	427.5 ± 6.1	410.3 ± 6.8	17.2 ± 5.9	449.4 ± 6.3	417.6 ± 5.5	31.8 ± 3.5	*

Note: Asterisks indicate significant differences between baseball and soccer players for the interocular difference (* *p* < 0.05 and ** *p* < 0.01).

## Data Availability

The datasets for this study can be found on OSF at https://osf.io/ed8b5/ (accessed on 28 January 2025).

## Supplementary Materials

The following supporting information can be downloaded at: https://www.mdpi.com/article/10.3390/vision9010009/s1.
